# Facile preparation of Fe_3_O_4_@Pt nanoparticles as peroxidase mimics for sensitive glucose detection by a paper-based colorimetric assay

**DOI:** 10.1098/rsos.220484

**Published:** 2022-09-28

**Authors:** Ye He, Panlin Wang, Xiaojing Chen, Yahuang Li, Jiajun Wei, Guoxi Cai, Kiyoshi Aoyagi, Wenxiang Wang

**Affiliations:** ^1^ Department of Health Inspection and Quarantine, School of Public Health, Fujian Medical University, Fuzhou, Fujian, People's Republic of China; ^2^ Fujian Province Key Laboratory of Environment and Health, School of Public Health, Fujian Medical University, Fuzhou, Fujian, People's Republic of China; ^3^ Department of International Health and Medical Anthropology, Institute of Tropical Medicine (NEKKEN), Nagasaki University, Nagasaki 852-8523, Japan; ^4^ Department of Public Health, Nagasaki University Graduate School of Biomedical Sciences, Nagasaki 852-8523, Japan

**Keywords:** Fe_3_O_4_@Pt NPs, nanoenzyme, glucose, colorimetric, paper-based device

## Abstract

A simple strategy to rapidly detect glucose was developed by utilizing core (Fe_3_O_4_)–shell (Pt) magnetic nanoparticles (Fe_3_O_4_@Pt NPs) as a nanoenzyme and a paper-based colorimetric sensor. In the presence of H_2_O_2_, Fe_3_O_4_@Pt NPs catalyze the redox reaction of 3,3′,5,5′-tetramethylbenzidine (TMB) and generate a colour change from colourless to blue. On this basis, a colorimetric glucose sensing method assisted by glucose oxidase (GOx) was developed. Under the optimal conditions, the detection limits of the proposed assay for H_2_O_2_ and glucose were 0.36 µM and 1.27 µM, respectively. Furthermore, the fabricated colorimetric method was successfully applied to analyze glucose concentrations by using a paper device as a measuring platform without a spectrometer. In addition, this method exhibited satisfactory recovery for glucose detection in human serum samples and urine samples, which satisfied the requirements for normal detection of real samples. This study provides a good candidate for health monitoring of glucose and also expands the applications of nanoenzymes and paper-based colorimetric assays in point-of-care testing.

## 1. Introduction

Diabetes is a chronic disease associated with various terrible complications, such as blindness, kidney failure, heart attacks, strokes and lower limb amputations [1]. Hence, it is vital to monitor and control the blood sugar level and urine sugar level to prevent and control the occurrence of diabetes and its complications. It is therefore very important to design a simple, convenient and easy-to-operate glucose sensor, which has become the centre of attention for researchers, clinicians and patients [2,3]. Scholars have developed a variety of methods for the determination of glucose [4], including electrochemistry [5–8], colorimetry [9–13], fluorimetry [14–16], chemiluminescence [17,18] and surface-enhanced Raman spectroscopy [19–22]. Among these techniques, colorimetric techniques have attracted considerable attention owing to their simple operation, ease of use, high visual sensitivity and low cost. The typical colorimetric biosensor for glucose detection is designed based on the activity of biological enzymes [23,24]. Nevertheless, the catalytic activities of natural enzymes are easily inhibited, and the enzymes are digested by proteases, which results in poor stability and reduced accuracy, cannot better match the simplicity and convenience of real-time detection, and limits their application. Therefore, a method instead of natural enzymes is needed for the detection of glucose, particularly in the application of point-of-care testing.

Nanoenzymes are artificial nanomaterials that can imitate the activities of natural enzymes [25–27]. Compared to natural enzymes, nanoenzymes have many irreplaceable advantages, such as high stability, low cost, adjustable catalytic activity and convenient modification [28–30]. It has been found that Fe_3_O_4_ nanoparticles have inherent peroxidase-like activity [31,32]. The catalytic mechanism of Fe_3_O_4_ might be explained by a ping-pong reaction mechanism. Fe_3_O_4_ could combine with the first substrate H_2_O_2_ to generate intermediate •OH, which could catch hold of one H^+^ from the hydrogen donor such as TMB [33]. Platinum is one of the most ideal shells with which to protect Fe_3_O_4_ nanoparticles from damage and aggregation [34]. Platinum (Pt) is a transition metal exhibiting chemical inertness and stability in air or a humid environment [35], and Pt NPs also have peroxidase-like activity [36–38]. This may be caused by the base-like decompositions of H_2_O_2_ on the surfaces of Pt NPs [33]. Fe_3_O_4_@Pt hybrid nanoparticles make full use of these two materials (precious metal and magnetic material) [39] and show better catalytic performance than individual metals through synergistic effects [40].

In this research, we constructed a simple approach to detect glucose by preparing uniformly dispersed core (Fe_3_O_4_)–shell (Pt) magnetic nanoparticles (Fe_3_O_4_@Pt NPs) as peroxidase mimetics, with which a paper-based colorimetric sensor is used. The strong colorimetric signal that appears on the paper is sufficient to distinguish normal (healthy) and hyperglycemic (diabetes) concentrations with the naked eye. The experimental method has the advantages of simple preparation and environmental protection and has broad prospects for application for rapid and timely detection of glucose.

## 2. Material and methods

### 2.1. Materials and apparatus

Glucose, sucrose, fructose, lactose and maltose were obtained from Macklin, Inc. (Shanghai, China). Chloroplatinic acid (H_2_PtCl_6_·6H_2_O) and 3,3′,5,5′-tetramethylbenzidine (TMB) were purchased from Aladdin Chemistry Co., Ltd. (Shanghai, China). Ferric chloride hexahydrate (FeCl_3_·6H_2_O), anhydrous sodium acetate, ethylene glycol (EG), sodium borohydride (NaBH_4_), sodium citrate dihydrate, 30% H_2_O_2_ and absolute ethanol were supplied by Sinopharm Chemical Reagent Co., Ltd. (Shanghai, China). Tween 20 was obtained from Sigma–Aldrich Co., Ltd. (Shanghai, China). All chemicals were of analytical grade and used without further purification. The water used in this experiment was purified with a Milli-Q water system (18.2 M*Ω*/cm).

Serum and urine were collected by the Fujian Maternal and Child Health Hospital, Affiliated Hospital of Fujian Medical University. The *in vitro* experimental protocol was approved by the Ethics Committee of Fujian Medical University (approval number: 2019021; approval date: March 8, 2019), and the volunteers provided consent.

X-ray diffraction (XRD) patterns of the products were obtained on an Ultima IV multipurpose X-ray diffraction system (Japan). X-ray photoelectron spectroscopy (XPS) was conducted with a Thermo Scientific K-Alpha spectrometer (Thermo Ltd. USA). Transmission electron microscopy (TEM) was performed on an FEI Tecnai G2 F20 (FEI Co. Ltd. USA) by placing a drop of sample solution on a TEM copper grid. Energy dispersive spectrometry (EDS) was used to determine the element ratios of iron and platinum. Fourier transform infrared (FTIR) spectra were obtained from a Fourier transform infrared spectrometer (Bruker VERTEX 70 & ALPHA, Bruker Ltd. Germany). Magnetic measurements were performed using a 7404 vibrating sample magnetometer (Lake Shore, Ltd., USA). The hydrodynamic sizes and zeta potentials of particles were measured on a Malvern Zetasizer ZEN 3700 (Malvern Panalytical Ltd., U.K.). UV–vis spectra and time-dependent absorbance changes were collected on an Infinite 200 Pro spectrophotometer (Tecan Ltd., Austria).

### 2.2. Synthesis of nanoenzymes

Fe_3_O_4_ NPs were synthesized by a reported one-step hydrothermal method [41] with minor modifications. In brief, 1.0 g of FeCl_3_·6H_2_O was dissolved in 35 ml of glycol in a 100-ml flask, and 0.415 g of sodium citrate dihydrate and 2.4 g of anhydrous sodium acetate were added. After all reactants were dissolved by vigorous stirring, the solution was transferred to a 50-ml stainless-steel autoclave lined with Teflon and heated to 200°C for 10 h. After cooling, the obtained black precipitate was washed with water and ethanol 6 times and dried in a vacuum oven at 50°C for 10 h.

Fe_3_O_4_@Pt NPs were synthesized. 12 ml of 1 mg ml^−1^ Fe_3_O_4_ NPs, 200 µl of 2% H_2_PtCl_6_·6H_2_O and 38 ml of water were stirred together under a nonmagnetic agitator in darkness for 30 min. Then, 500 µl of freshly prepared 0.5 mg ml^−1^ NaBH_4_ was added. After stirring for 8 min, 0.2 g of sodium citrate dihydrate was added to the solution with a further 5 min of stirring. The resulting brown–black solution was washed with water 6 times. The obtained Fe_3_O_4_@Pt NPs were stored at 4°C.

### 2.3. Kinetic parameters

The absorption spectra at intervals of 60 s were recorded by a multi-mode absorbance microboard reader (TECAN, Infinite 200 Pro), and the reaction kinetics of the catalytic oxidation of TMB were studied. Unless otherwise stated, the reaction was performed at room temperature. 50 µl 20 µg ml^−1^ Fe_3_O_4_ NPs or Fe_3_O_4_@Pt NPs were added to 100 µl of HAc-NaAc buffer (2 mM, pH 4.0) in the presence of different concentrations of TMB or H_2_O_2_.

### 2.4. Hydrogen peroxide detection

In colorimetric experiments for the detection of H_2_O_2_, 100 µl of 1.6 mM TMB, 50 µl of 20 µg ml^−1^ Fe_3_O_4_@Pt NPs, 100 µl of HAc-NaAc buffer (2 mM, pH 4.0) and 100 µl of different concentrations of H_2_O_2_ solutions (0.005–1 mM) were sequentially added to the vials. Then, the mixed solution was incubated at 35°C for 30 min. The colour change of the solution was observed by the naked eye or measured by a microplate reader.

To verify the long-term storage stability of Fe_3_O_4_@Pt NPs, we measured the colour change of the solution by the detection of 1 mM H_2_O_2_ using the above method every two days for 30 days.

### 2.5. Glucose detection

For glucose determination, 100 µl of different concentrations of glucose were first incubated with 40 µl of 2.5 kU ml^−1^ glucose oxidase (GOx) in PBS (137 mM NaCl, 2.7 mM KCl, 2 mM KH_2_PO_4_, 10 mM Na_2_HPO_4_, pH 7.4) at 37°C for 30 min. After that, 50 µl of 20 µg ml^−1^ Fe_3_O_4_@Pt NPs, 100 µl of 1.6 mM TMB and 100 µl of HAc-NaAc buffer were added to the glucose reaction solution and incubated at 35°C for 30 min. The spectra of the final solution were recorded by a microplate reader.

### 2.6. Preparation of a paper-based platform

First, the filter paper was cut into small discs of 1 cm in diameter. Then, 5 µl of glucose and 5 µl of 2.5 kU ml^−1^ GOx were dropped on filter paper and allowed to react for 30 min, and 5 µl of 20 µg ml^−1^ Fe_3_O_4_@Pt NPs and 5 µl of 1.6 mM TMB were added. After reaction for 10 min, colour images were captured by camera, and grayscale intensity analysis was performed with ImageJ software.

## 3. Results and discussion

### 3.1. Detection principle

**Scheme 1.**
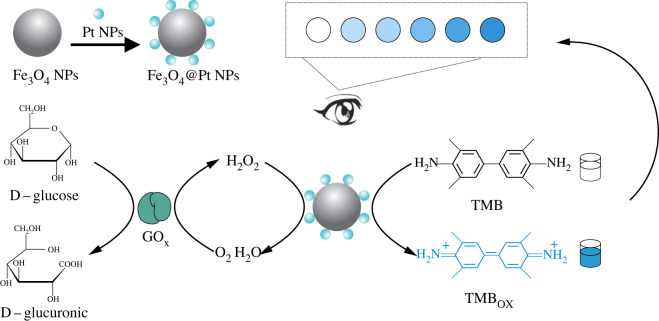
Construction of Fe_3_O_4_@Pt NPs and the H_2_O_2_ and glucose detection assay using Fe_3_O_4_@Pt NPs.

Both Fe_3_O_4_ NPs and Pt NPs have inherent peroxidase-like activity and show better catalytic performance than the individual metals alone because of a synergistic effect, and they effectively catalyze the oxidation of TMB by hydrogen peroxide to produce a colour change [42]. The degree of colour rendered by TMB was proportional to the concentration of H_2_O_2_. The detailed principle of this method is shown in scheme 1. In solution, glucose was first oxidized by glucose oxidase (GOx) to form H_2_O_2_. By adding Fe_3_O_4_@Pt NPs and TMB solution, Fe_3_O_4_@Pt NPs effectively catalyzed the oxidation of TMB by H_2_O_2_, causing TMB to change from colourless to blue in the solution, which provided a sensing platform for visual detection of H_2_O_2_ and glucose.

### 3.2. Characterization of Fe_3_O_4_ NPs and Fe_3_O_4_@Pt NPs

**Figure 1.**
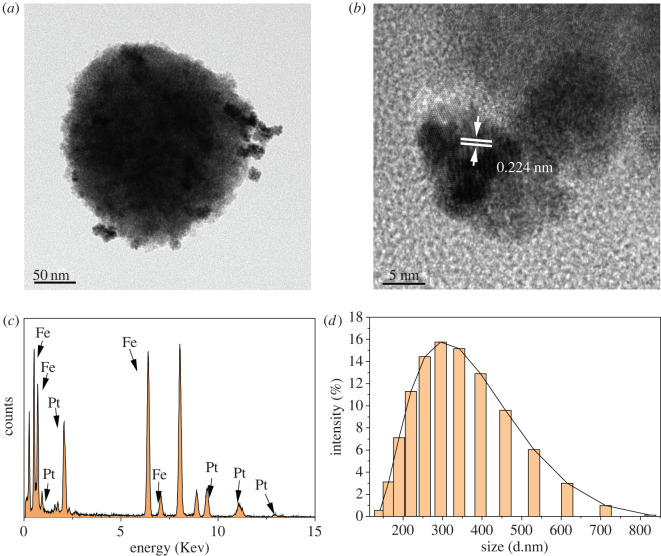
(*a*) Low- and (*b*) high-magnification TEM images of Fe_3_O_4_@Pt NPs. (*c*) EDS spectra of Fe_3_O_4_@Pt NPs. (*d*) Size distribution of Fe_3_O_4_@Pt NPs.

TEM images and particle size distribution curves for the prepared Fe_3_O_4_ NPs and Fe_3_O_4_@Pt NPs are shown in electronic supplementary material, figure S1 and figure 1. As shown in electronic supplementary material, figure S1A, B, Fe_3_O_4_ NPs were spherical, the mean particle size was 239.8 nm (electronic supplementary material, figure S1D). The nanoparticles were polycrystalline clusters with a lattice fringe spacing of 0.48 nm (electronic supplementary material, figure S1B), which was consistent with the (111) crystal plane of the cubic spinel structure. Figure 1*a,b* shows TEM data for Fe_3_O_4_@Pt NPs, in which the core and shell components could easily be distinguished by the difference in brightness. The Fe_3_O_4_@Pt NPs were indeed monodisperse spherical particles with a mean particle size of 281.3 nm (figure 1*d*). The lattice fringe spacing was 0.224 nm (figure 1*b*), which was consistent with the crystalline plane of Pt. Figure 1*b* shows that the Pt NPs were uniformly distributed on the core of the Fe_3_O_4_ NPs, and the diameter of the Pt NPs was approximately 7.75 nm. According to the chemical compositions of randomly selected Fe_3_O_4_ NPs and Fe_3_O_4_@Pt NPs analysed by energy dispersive spectrometry, the atomic ratio of Fe:Pt was 1.95:1 (electronic supplementary material, figure S1C and figure 1*c*).

**Figure 2.**
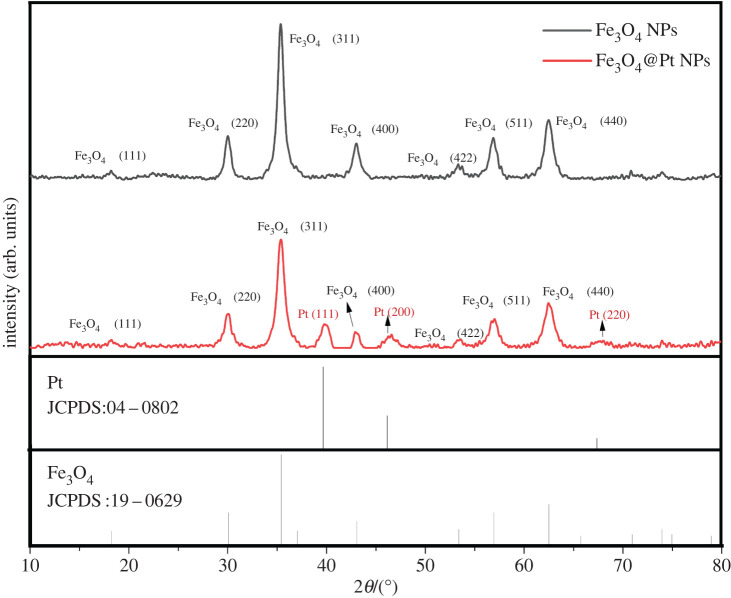
XRD patterns of Fe_3_O_4_ NPs and Fe_3_O_4_@Pt NPs.

The XRD patterns of Fe_3_O_4_ NPs and Fe_3_O_4_@Pt NPs are shown in figure 2. The diagram shows diffraction peaks at 2*θ* = 18.66°, 30.21°, 35.52°, 43.27°, 53.64°, 57.06° and 62.70°, which correspond to the (111), (220), (311), (400), (422), (511) and (440) planes, respectively, indicating that the sample was highly crystalline Fe_3_O_4_ NPs with a face centered cubic (FCC) structure (JCPDS 19-0629) for the spinel structure. The diffraction peaks at 2*θ* = 39.78°, 46.35° and 67.48° corresponded to the (111), (200) and (220) planes, respectively, of Pt (JCPDS 04-0802). The XRD results confirmed the successful synthesis of Fe_3_O_4_@Pt NPs.

**Figure 3.**
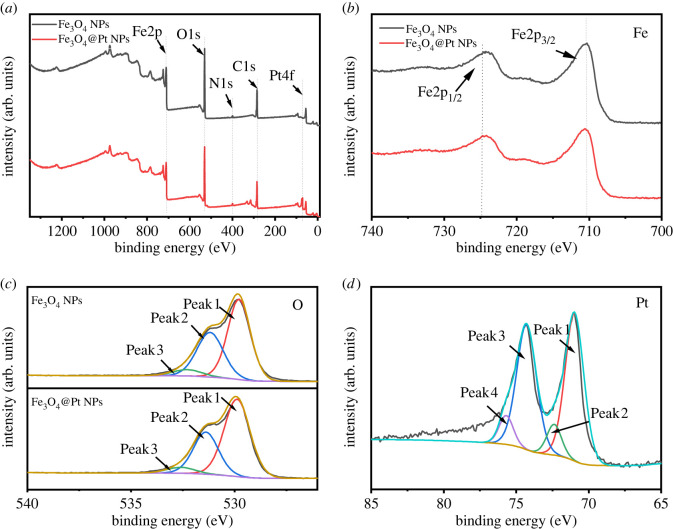
(*a*) XPS spectra of Fe_3_O_4_ NPs and Fe_3_O_4_@Pt NPs. High resolution peak-fitting XPS spectra of (*b*) Fe 2p, (*c*) O 1s, and (*d*) Pt 4f.

The XPS spectra of Fe_3_O_4_ NPs and Fe_3_O_4_@Pt NPs are shown in figure 3. Figure 3*a* shows the full XPS spectra for the Fe_3_O_4_ NPs and Fe_3_O_4_@Pt NPs. The main elements on the surface of the sample were Fe, O, C, N and Pt. XPS results for the Fe_3_O_4_ NPs and Fe_3_O_4_@Pt NPs catalysts are shown in figure 3*b–d*. In the Fe 2p X-ray photoelectron spectrum of Fe_3_O_4_ (figure 3*b*), the peaks at 711.2 eV and 724.6 eV could be attributed to Fe 2p_3/2_ and Fe 2p_1/2_, respectively, which indicates that Fe_3_O_4_ NPs were the source of Fe, which was very close to the value of Fe_3_O_4_ published in the literature [43]. The O 1s spectra of Fe_3_O_4_ NPs and Fe_3_O_4_@Pt NPs could be divided into three peaks (figure 3*c*). The O 1s spectrum of Fe_3_O_4_ NPs has a maximum peak at 529.8 eV, which belongs to the Fe-O bond [44]. Peak 2 (≈531.2 eV) was attributed to OH groups on the surface of Fe_3_O_4_ and/or oxygen in the oxygen vacancy, and peak 3 (≈532.3 eV) indicated adsorption of H_2_O from air on the surface of the Fe_3_O_4_ NPs carrier.

Figure 3*d* shows the Pt 4f XPS spectrum of Fe_3_O_4_@Pt NPs. After curve fitting, the spectrum consisted of two pairs of peaks: peak 1 ≈ 71.0 eV, peak 3 ≈ 74.3 eV, peak 2 ≈ 72.4 eV and peak 4 ≈ 75.7 eV, which belong to Pt(0) and Pt^2+^, respectively [45]. The XPS results were consistent with those from TEM, XRD and EDS mapping.

**Figure 4.**
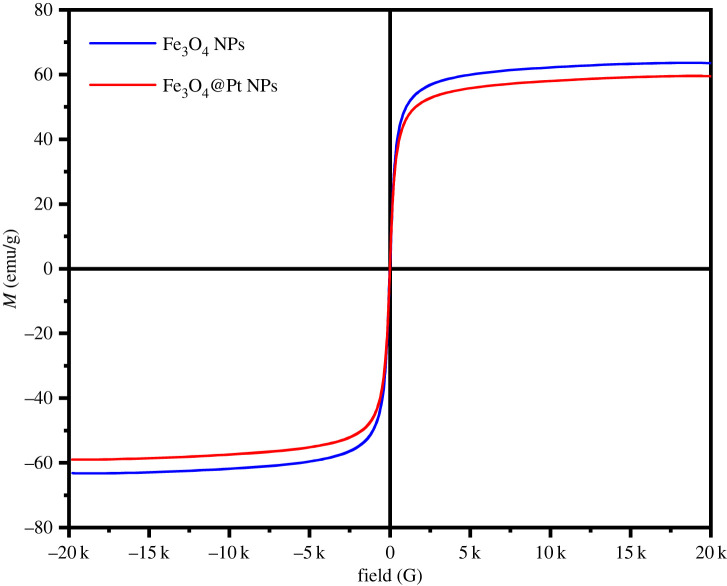
Magnetic hysteresis curves of Fe_3_O_4_ NPs and Fe_3_O_4_@Pt NPs.

Figure 4 shows the hysteresis loops of Fe_3_O_4_ NPs and Fe_3_O_4_@Pt NPs measured at room temperature. These results showed that the hysteresis loops had almost no hysteresis and coercivity. The test results showed that the saturation magnetizations of Fe_3_O_4_ NPs and Fe_3_O_4_@Pt NPs were 63.61 emu/g and 59.57 emu g^−1^, respectively, which showed that binding of Pt NPs had little effect on the magnetic properties. It was reported in the literature that the magnetic particles showed typical superparamagnetism because the particles were composed of ultrafine magnetite nanocrystals [46]. Under the action of an external magnetic field, the prepared Fe_3_O_4_@Pt magnetic particles actively responded to the magnetic field and were attracted by the magnetic field; however, once the external magnetic field was withdrawn, the particles themselves had no residual magnetism. This superparamagnetism is very important for magnetic separation and the manufacture of renewable enzyme reactors.

### 3.3. Feasibility analysis and kinetic analysis

**Figure 5.**
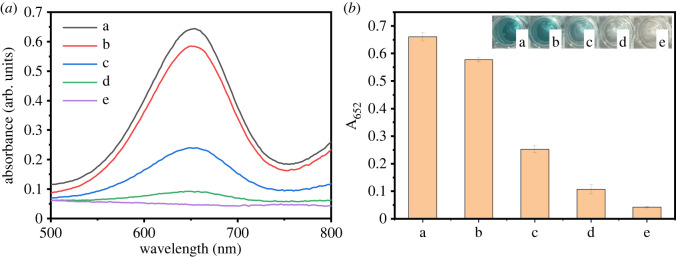
UV–vis absorption spectra (the inset shows the corresponding colorimetric photographs) of sodium citrate buffer (pH 4.0) containing 1 mM H_2_O_2_ and 1.6 mM TMB in the presence of Fe_3_O_4_@Pt NPs (20 µg ml^−1^) (*a*), Fe_3_O_4_ NPs (20 µg ml^−1^) (*b*) and Pt NPs (20 μg ml^−1^) (*c*). The control group constituted the same reaction system but without TMB (*d*) and H_2_O_2_ (*e*).

To verify the inherent catalytic potential of Fe_3_O_4_@Pt NPs in catalysis, the following experiments were performed. As shown in figure 5, when there was no TMB or H_2_O_2_, the solution was almost colourless, and the absorbance in the measured range was very low. When TMB and H_2_O_2_ were present at the same time, the Fe_3_O_4_@Pt NPs solution, Pt NPs solution (the synthesis method of Pt NPs was based on the literature method [47]) and Fe_3_O_4_ NPs solution were blue (as shown in the illustration of figure 5*b*), the absorption peak was at 652 nm, and the absorbance intensity changed obviously. The peroxidase-like activity of Fe_3_O_4_@Pt NPs was 1.2 times and 2.6 times stronger than that of Pt NPs and Fe_3_O_4_ NPs, respectively. Such catalytic enhancement could be attributed to a synergetic effect that occurred at the interfaces of Pt NPs and the Fe_3_O_4_ NPs that support the heterostructure.

To further study the kinetics of Fe_3_O_4_@Pt NPs catalysis, the steady-state kinetic parameters (*K*_m_ and *V*_max_) of Fe_3_O_4_@Pt NPs in reactions with H_2_O_2_ or TMB substrates were determined. As shown in electronic supplementary material, figure S2, when using TMB or H_2_O_2_ as a substrate, the enzyme activity conformed to typical Michaelis–Menten kinetics. In addition, Michaelis–Menten curves (electronic supplementary material, figure S2A, C) and Lineweaver-Burk diagrams (electronic supplementary material, figure S2B, D) for H_2_O_2_ and TMB were obtained over a certain concentration range. In addition, the initial maximum reaction rate (*V_max_*) and Michaelis constant (*K_m_*) of the Fe_3_O_4_@Pt hybrid NPs were calculated by using the double reciprocal Lineweaver-Burk diagram based on the following function:

$$\frac{1}{V}=\frac{K_{m}}{V_{\max}}\times \frac{1}{\left[C\right]}+\frac{1}{V_{\max}},$$

where *V* is the initial velocity, and [*C*] is the substrate concentration. *K*_m_ is a well-known important index for catalytic materials and can be used to determine the catalytic activity and affinity between enzymes and substrates. Electronic supplementary material, figure S2C, D shows the catalytic activities of H_2_O_2_ and TMB substrates and their corresponding double reciprocal curves. The kinetic parameters, including the Michaelis constant (*K*_m_) and maximum reaction rate (*V*_max_), were obtained from the double reciprocal plot. Generally, a smaller *Km* corresponds to stronger affinity between enzyme and substrate. As shown in electronic supplementary material, table S1, the *K*_m_ value for the reaction of Fe_3_O_4_@Pt NPs (95.6 mM) with H_2_O_2_ was higher than that of HRP (3.7 mM), which indicates that the affinity of Fe_3_O_4_@Pt NPs for H_2_O_2_ was weaker than that of HRP, and Fe_3_O_4_@Pt NPs require more H_2_O_2_ to depict the same peroxidase activity as HRP. Fe_3_O_4_@Pt NPs (0.2 mM) and TMB had similar *K_m_* values to HRP (0.4 mM), which indicates that the affinity of Fe_3_O_4_@Pt NPs for TMB was close to that of HRP. The obtained results were identical to those in the literature [48].

### 3.4. Optimization of experimental conditions

**Figure 6.**
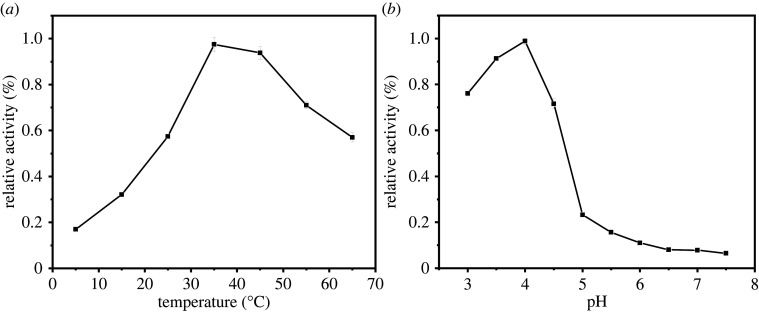
Influence of temperature (*a*) and pH (*b*) on the enzyme activity for a solution containing 1 mM H_2_O_2_ and 1.6 mM TMB and Fe_3_O_4_@Pt NPs (20 µg ml^−1^).

To better optimize the catalytic performance of the Fe_3_O_4_@Pt NPs reactions, the catalytic activity was studied at different pH values and temperatures. As shown in figure 6, the relative activity of Fe_3_O_4_@Pt NPs increased when the temperature increased from 20°C to 35°C and decreased when the temperature increased to 65°C (figure 6*a*), which indicates that the optimal temperature for nanoparticles is 35°C. The decrease in peroxidase-like activity might be due to the morphological change/losses of Fe_3_O_4_@Pt NPs at high temperature or the accelerated decomposition rate of H_2_O_2_ to O_2_ and H_2_O. Meanwhile, the relative activity of Fe_3_O_4_@Pt NPs increased when the pH was increased from 3.0 to 4.0 and decreased from pH 4.0 to 7.5 (figure 6*b*). Therefore, the optimal pH for the activity of the separated nanoparticles is pH 4.0. Therefore,35°C and pH 4.0 were selected for the subsequent Fe_3_O_4_@Pt NPs analysis.

### 3.5. Stability test

**Figure 7.**
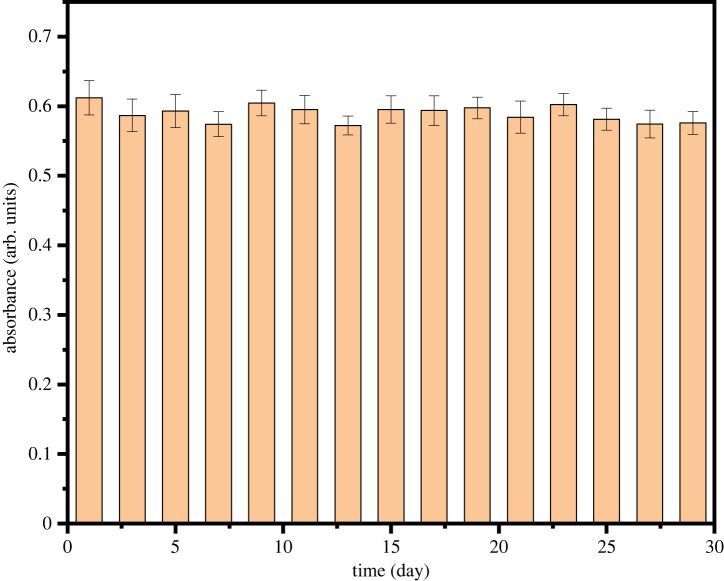
Stability tests of Fe_3_O_4_@Pt NPs.

Figure 7 shows the long-term storage stability of Fe_3_O_4_@Pt NPs. No apparent changes were observed in the absorption peak at 652 nm within 30 days, which indicates that Fe_3_O_4_@Pt NPs have good long-term storage stability.

### 3.6. Specificity test

**Figure 8.**
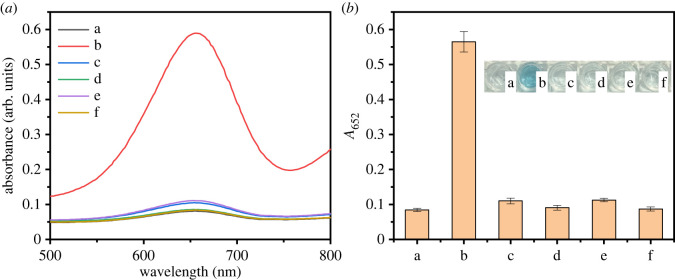
Responses of the developed Fe_3_O_4_@Pt NP-based analytical methods to blank samples (*a*) and various sugars, including glucose (*b*), fructose (*c*), lactose (*d*), maltose (*e*) and sucrose (*f*) (the inset shows the corresponding colorimetric photographs).

To further study the specificity of this method, blank samples, sucrose, fructose, lactose and maltose were selected for control experiments. As shown in figure 8, even if the concentration of the control sample was five times the glucose concentration, the glucose-containing sample had much higher absorbance than the control sample. In addition, the blue changes for glucose samples could be observed with the naked eye in the sucrose, fructose, lactose and maltose samples (as shown in the illustration of figure 8*b*). The experimental results show that the colorimetric reaction system had high selectivity for glucose.

### 3.7. H_2_O_2_ and glucose assay based on the Fe_3_O_4_@Pt NPs

Based on the optimized conditions, the colorimetric detection system for H_2_O_2_ and glucose was constructed using the Fe_3_O_4_@Pt NPs as a catalyst. Electronic supplementary material, figure S3A shows the corresponding changes in absorbance at 652 nm when the H_2_O_2_ concentration was varied from 5–1000 µM. Electronic supplementary material, figure S3B shows that the linear range was 5–400 µM. The limits of detection (LOD) in this work were calculated as 3 *σ*/*S*, where *σ* is the standard deviation of replicate measurements of the blank sample signal and *S* is the sensitivity (slope of the regression equation). The LOD was 0.36 µM based on the hydrogen peroxide detection method. The detection limits of different nanoenzymes for colorimetric hydrogen peroxide detection are listed in electronic supplementary material, table S2. The limits for the detection of hydrogen peroxide by nanoenzymes were comparable to those in the literature and had been improved.

**Figure 9.**
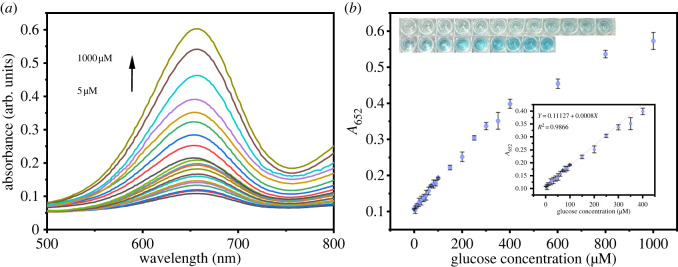
(*a*) UV–vis spectra to show the change in glucose concentration and (*b*) dose–response curve for the glucose concentration and absorbance (the inset shows the corresponding homology calibration curve and colorimetric photographs).

Figure 9*a* shows the changes in absorbance at 652 nm when the glucose concentration was varied from 5–1000 µM. Figure 9*b* shows that the linear range was 5–400 µM, and the detection limit was 1.27 µM. The detection limits of different nanoenzymes for colorimetric glucose detection are listed in electronic supplementary material, table S3. By comparison, the LOD of hydrogen peroxide by nanoenzymes were comparable to those in the literature and had been improved.

### 3.8. Detection of blood glucose and urine glucose

To verify the practical application value of the system, glucose in diluted serum and urine was detected with Fe_3_O_4_@Pt NPs. In tables 1 and 2, the recoveries of the measured values based on the standard curve were 92.76–102.93% and 91.81–103.48%, respectively, which indicates that the detection system exhibited good detection of glucose in actual samples.

**Table 1.** Detection of glucose in human blood samples. (*n* = 3)

glucose added (*μ*M)	glucose found (*μ*M)	recovery (%)	RSD (%)
0	—	—	—
20	18.98	94.90	2.25
50	47.00	94.00	1.65
80	74.21	92.76	0.83
100	99.99	99.99	0.77
200	190.4	95.24	1.42
400	411.7	102.93	1.95

**Table 2.** Detection of glucose levels in human urine. (*n* = 3)

glucose added (*μ*M)	glucose found (*μ*M)	recovery (%)	RSD (%)
0	—	—	—
20	18.36	91.81	0.37
50	48.61	97.23	0.71
80	76.64	95.80	1.26
100	94.95	94.95	0.57
200	191.8	95.93	2.28
400	413.9	103.48	2.06

### 3.9. Paper-based detection of glucose

**Figure 10.**
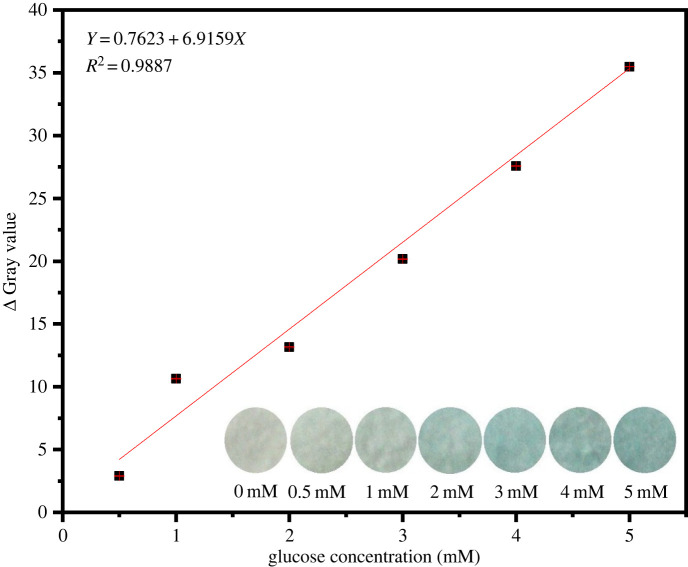
Greyscale value of the glucose concentration change (the inset shows the corresponding photographs).

The use of test strips to detect glucose was also studied. The sensing efficiency was determined for a paper-based sensor strip. Low-cost and easy-to-use cellulose filter paper was employed in the study. As shown in the inset of figure 10, the colour of the dye strip ranged from colourless to blue, and the colour depth increased with increasing glucose concentration. Figure 10 illustrates the relationship between grayscale value and glucose concentration. It shows a good linear relationship, the linear range was 0.5–5 mM (*R*^2^ = 0.9887), which satisfies the requirements for normal detection of human serum glucose (3.9–6.4 mM), and the detection limit was 0.39 mM. Hence, the Fe_3_O_4_@Pt NPs and paper-based method developed in this work can be applied to detect glucose without requiring a measuring instrument.

To study the application of this paper-based platform in studies of real samples, we detected the peak values for glucose concentration in diluted serum and urine at different concentrations and compared them with those of a commercial blood glucose metre. As shown in tables 3 and 4, the recoveries of the measured values based on the standard curve for the detection of human blood glucose and human urine glucose were 97.14–99.62% and 97.04–104.93%, respectively. In addition, the results of this method were consistent with those of the blood glucose meter sold in the market. The results showed that the developed system had a good effect on the detection of glucose in actual samples.

**Table 3.** Detection of glucose in human blood samples. (*n* = 3)

glucose added (mM)	colour	glucose found (mM)	recovery (%)	RSD (%)	glucose metre
0	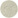	—	—	—	—
1	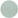	0.9882	98.82	4.86	—
3	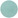	2.9886	99.62	1.11	2.8
5	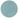	4.8569	97.14	1.99	4.9

**Table 4.** Detection of glucose levels in human urine. (*n* = 3)

glucose added (mM)	colour	glucose found (mM)	recovery (%)	RSD (%)	glucose metre
0	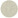	—	—	—	—
1	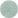	0.9730	97.30	4.55	—
3	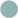	3.148	104.93	2.88	2.8
5	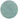	4.852	97.04	4.26	4.8

## 4. Conclusion

In summary, a Pt nanoparticle composite catalyst grown *in situ* on Fe_3_O_4_ NPs was constructed to detect H_2_O_2_ and glucose. Due to a synergistic effect, the core (Fe_3_O_4_)–shell (Pt) magnetic nanoparticles (Fe_3_O_4_@Pt NPs) had higher catalytic activity than the Fe_3_O_4_ NPs. The material was applied to detect glucose in serum and urine samples. The linear ranges for H_2_O_2_ and glucose were 5–400 µM (*R*^2^ = 0.9968) and 5–400 µM (*R*^2^ = 0.9866), respectively, and the detection limits were 0.36 µM and 1.27 µM, respectively. The recoveries with serum and urine were 92.76 − 102.93% and 91.81 − 103.48%, respectively. Furthermore, a fabricated colorimetric method was successfully applied to analyze glucose concentrations using a paper device as a measuring platform without requiring a spectrometer. This method exhibited satisfactory recovery values for glucose detection in human serum samples and urine samples and satisfies the requirements for normal detection in real samples. This study demonstrates a good candidate for the health monitoring of glucose and expands the applications of nanoenzymes and paper-based colorimetric assays in point-of-care testing.

## Data Availability

The datasets supporting this article have been uploaded as part of the electronic supplementary material [49].

## References

[1] Boles A, Kandimalla R, Reddy PH. 2017 Dynamics of diabetes and obesity: epidemiological perspective. Biochim. Biophys. Acta Mol. Basis Dis. **1863**, 1026-1036. (10.1016/j.bbadis.2017.01.016) 28130199 PMC5429876

[2] Yun J et al. 2009 Glucose deprivation contributes to the development of KRAS pathway mutations in tumor cells. Science **325**, 1555-1559. (10.1126/science.1174229) 19661383 PMC2820374

[3] Maric T et al. 2019 Bioluminescent-based imaging and quantification of glucose uptake in vivo. Nat. Methods. **16**, 526-532. (10.1038/s41592-019-0421-z) 31086341 PMC6546603

[4] Oliver NS, Toumazou C, Cass AE, Johnston DG. 2009 Glucose sensors: a review of current and emerging technology. Diabet Med. **26**, 197-210. (10.1111/j.1464-5491.2008.02642.x) 19317813

[5] Hassan MH, Vyas C, Grieve B, Bartolo P. 2021 Recent Advances in enzymatic and non-enzymatic electrochemical glucose sensing. Sensors **21**, 4672. (10.3390/s21144672) 34300412 PMC8309655

[6] Zhou Y, Hu Q, Yu F, Ran GY, Wang HY, Shepherd ND, D'Alessandro DM, Kurmoo M, Zuo JL. 2020 A metal-organic framework based on a nickel bis(dithiolene) connector: synthesis, crystal structure, and application as an electrochemical glucose sensor. J. Am. Chem. Soc. **142**, 20 313-20 317. (10.1021/jacs.0c09009) 33185447

[7] Xu J, Sun Y, Zhang J. 2020 Solvothermal synthesis of Fe_3_O_4_ nanospheres for high-performance electrochemical non-enzymatic glucose sensor. Sci. Rep. **10**, 16026. (10.1038/s41598-020-73090-4) 32994458 PMC7524729

[8] Li Y, Cai R, Lü R, Gao L, Qin S. 2018 Template synthesis of the Cu_2_O nanoparticle-doped hollow carbon nanofibres and their application as non-enzymatic glucose biosensors. R. Soc. Open Sci. **5**, 181474. (10.1098/rsos.181474) 30662752 PMC6304140

[9] Zhao Y, Yang J, Shan G, Liu Z, Cui A, Wang A, Chen Y, Liu Y. 2020 Photothermal-enhanced tandem enzyme-like activity of Ag_2−x_Cu_x_S nanoparticles for one-step colorimetric glucose detection in unprocessed human urine. Sens. Actuators, B. **305**, 127420. (10.1016/j.snb.2019.127420)

[10] Zhang J, Dai X, Song Z-L, Han R, Ma L, Fan G-C, Luo X. 2020 One-pot enzyme- and indicator-free colorimetric sensing of glucose based on MnO_2_ nano-oxidizer. Sens. Actuators, B. **304**, 127304. (10.1016/j.snb.2019.127304)

[11] Kim MS, Kim DH, Lee J, Ahn HT, Kim MI, Lee J. 2020 Self color-changing ordered mesoporous ceria for reagent-free colorimetric biosensing. Nanoscale **12**, 1419-1424. (10.1039/c9nr09182c) 31909409

[12] Phiri MM, Mulder DW, Vorster BC. 2019 Seedless gold nanostars with seed-like advantages for biosensing applications. R. Soc. Open Sci. **6**, 181971. (10.1098/rsos.181971) 30891302 PMC6408411

[13] Chen P, Zhong H, Wang X, Shao C, Zhi S, Li X-R, Wei C. 2019 A label-free colorimetric strategy for facile and low-cost sensing of ascorbic acid using MnO_2_ nanosheets. Anal. Methods **11**, 1469-1474. (10.1039/c9ay00091g)

[14] Cai Q, Meng H, Liu Y, Li Z. 2019 Fluorometric determination of glucose based on a redox reaction between glucose and aminopropyltriethoxysilane and in-situ formation of blue-green emitting silicon nanodots. Mikrochim. Acta. **186**, 78. (10.1007/s00604-018-3189-4) 30627875

[15] Ramos-Soriano J, Benitez-Benitez SJ, Davis AP, Galan MC. 2021 A vibration-induced-emission-based fluorescent chemosensor for the selective and visual recognition of glucose. Angew. Chem. Int. Ed. Engl. **60**, 16 880-16 884. (10.1002/anie.202103545) PMC8362141 33857348

[16] Mello GPC, Simões EFC, Crista DMA, Leitão JMM, Pinto da Silva L, Esteves da Silva JCG. 2019 Glucose sensing by fluorescent nanomaterials. Crit. Rev. Anal. Chem. **49**, 542-552. (10.1080/10408347.2019.1565984) 30739473

[17] Dang P, Liu X, Ju H, Wu J. 2020 Intensive and persistent chemiluminescence system based on nano-/bioenzymes with local tandem catalysis and surface diffusion. Anal. Chem. **92**, 5517-5523. (10.1021/acs.analchem.0c00337) 32195577

[18] Zhao Y, Xu X, Ma Y, Tan H, Li Y. 2020 A novel peroxidase/oxidase mimetic Fe-porphyrin covalent organic framework enhanced the luminol chemiluminescence reaction and its application in glucose sensing. Luminescence **35**, 1366-1372. (10.1002/bio.3899) 32533573

[19] Hu S, Jiang Y, Wu Y, Guo X, Ying Y, Wen Y, Yang H. 2020 Enzyme-free tandem reaction strategy for surface-enhanced Raman scattering detection of glucose by using the composite of Au nanoparticles and porphyrin-based metal-organic framework. ACS Appl. Mater. Interfaces **12**, 55 324-55 330. (10.1021/acsami.0c12988) 33228360

[20] Xu M, Zhang L, Zhao F. 2020 One-pot aqueous synthesis of icosahedral Au as bifunctional candidates for enhanced glucose electrooxidation and surface-enhanced Raman scattering. ACS Appl. Mater. Interfaces. **12**, 12 186-12 194. (10.1021/acsami.9b15715) 32054264

[21] Ju J et al. 2020 Surface enhanced Raman spectroscopy based biosensor with a microneedle array for minimally invasive in vivo glucose measurements. ACS Sens. **5**, 1777-1785. (10.1021/acssensors.0c00444) 32426978

[22] Sun D, Qi G, Xu S, Xu W. 2016 Construction of highly sensitive surface-enhanced Raman scattering (SERS) nanosensor aimed for the testing of glucose in urine. RSC Adv. **6**, 53 800-53 803.

[23] Steiner MS, Duerkop A, Wolfbeis OS. 2011 Optical methods for sensing glucose. Chem. Soc. Rev. **40**, 4805-4839. (10.1039/c1cs15063d) 21674076

[24] Xiong Y, Zhang Y, Rong P, Yang J, Wang W, Liu D. 2015 A high-throughput colorimetric assay for glucose detection based on glucose oxidase-catalyzed enlargement of gold nanoparticles. Nanoscale **7**, 15 584-15 588. (10.1039/c5nr03758a) 26360908

[25] Adeniyi O, Sicwetsha S, Mashazi P. 2020 Nanomagnet-silica nanoparticles decorated with Au@Pd for enhanced peroxidase-like activity and colorimetric glucose sensing. ACS Appl. Mater. Interfaces **12**, 1973-1987. (10.1021/acsami.9b15123) 31846292

[26] Liang M, Yan X. 2019 Nanozymes: from new concepts, mechanisms, and standards to applications. Acc. Chem. Res. **52**, 2190-2200. (10.1021/acs.accounts.9b00140) 31276379

[27] Jin X, Zhong Y, Chen L, Xu L, Wu Y, Fu F. 2018 A palladium-doped graphitic carbon nitride nanosheet with high peroxidase-like activity: preparation, characterization, and application in glucose detection. Part. Part. Syst. Charact. **35**, 1700359. (10.1002/ppsc.201700359)

[28] Yang W, Weng C, Li X, Xu W, Fei J, Hong J, Zhang J, Zhu W, Zhou X. 2022 An ‘on-off’ ratio photoluminescence sensor based on catalytically induced PET effect by Fe_3_O_4_ NPs for the determination of coumarin. Food Chem. **368**, 130838. (10.1016/j.foodchem.2021.130838) 34425336

[29] Li Y, Chang Y, Yuan R, Chai Y. 2018 Highly efficient target recycling-based netlike Y-DNA for regulation of electrocatalysis toward methylene blue for sensitive DNA detection. ACS Appl. Mater. Interfaces **10**, 25 213-25 218. (10.1021/acsami.8b08545) 29979026

[30] Chen P, Zhong H, Li X-R, Li M, Zhou S. 2021 Palygorskite@Co_3_O_4_ nanocomposites as efficient peroxidase mimics for colorimetric detection of H_2_O_2_ and ascorbic acid. Appl. Clay Sci. **209**, 106109. (10.1016/j.clay.2021.106109)

[31] Gao L et al. 2007 Intrinsic peroxidase-like activity of ferromagnetic nanoparticles. Nat. Nanotechnol. **2**, 577-583. (10.1038/nnano.2007.260) 18654371

[32] Wei H, Wang E. 2008 Fe_3_O_4_ magnetic nanoparticles as peroxidase mimetics and their applications in H_2_O_2_ and glucose detection. Anal. Chem. **80**, 2250-2254. (10.1021/ac702203f) 18290671

[33] Huang Y, Ren J, Qu X. 2019 Nanozymes: classification, catalytic mechanisms, activity regulation, and applications. Chem. Rev. **119**, 4357-4412. (10.1021/acs.chemrev.8b00672) 30801188

[34] He SB, Chen RT, Wu YY, Wu GW, Peng HP, Liu AL, Deng HH, Xia XH, Chen W. 2019 Improved enzymatic assay for hydrogen peroxide and glucose by exploiting the enzyme-mimicking properties of BSA-coated platinum nanoparticles. Mikrochim. Acta. **186**, 778. (10.1007/s00604-019-3939-y) 31728642

[35] Bao YW, Hua XW, Ran HH, Zeng J, Wu FG. 2019 Metal-doped carbon nanoparticles with intrinsic peroxidase-like activity for colorimetric detection of H_2_O_2_ and glucose. J. Biomed. Mater. Res. B Appl. Biomater. **7**, 296-304. (10.1039/c8tb02404a) 32254554

[36] He SB, Yang L, Lin XL, Peng HP, Lin Z, Deng HH, Chen W, Hong GL. 2020 Sodium alginate modified platinum nanozymes with highly efficient and robust oxidase-like activity for antioxidant capacity and analysis of proanthocyanidins. Front. Chem. **8**, 654. (10.3389/fchem.2020.00654) 32850667 PMC7419988

[37] Han Q, Wang X, Liu X, Xiao W, Cai S, Wang C, Yang R. 2019 Controllable fabrication of magnetic core-shell nanocomposites with high peroxide mimetic properties for bacterial detection and antibacterial applications. J. Biomed. Mater. Res. B Appl. Biomater. **7**, 1124-1132. (10.1039/c8tb02834f) 32254780

[38] Deng H, Liu H, Kang W, Lei C, Nie Z, Huang Y, Yao S. 2020 Biomineralization synthesis of a near-infrared fluorescent nanoprobe for direct glucose sensing in whole blood. Nanoscale **12**, 864-870. (10.1039/c9nr06691h) 31833533

[39] Yang Q, Li N, Li Q, Chen S, Wang HL, Yang H. 2019 Amperometric sarcosine biosensor based on hollow magnetic Pt-Fe_3_O_4_@C nanospheres. Anal. Chim. Acta. **1078**, 161-167. (10.1016/j.aca.2019.06.031) 31358215

[40] Lai X, Zhang G, Zeng L, Xiao X, Peng J, Guo P, Zhang W, Lai W. 2021 Synthesis of PDA-mediated magnetic bimetallic nanozyme and its application in immunochromatographic assay. ACS Appl. Mater. Interfaces. **13**, 1413-1423. (10.1021/acsami.0c17957) 33346647

[41] Li S, Zhao X, Yu X, Wan Y, Yin M, Zhang W, Cao B, Wang H. 2019 Fe_3_O_4_ nanozymes with aptamer-tuned catalysis for selective colorimetric analysis of ATP in blood. Anal. Chem. **91**, 14 737-14 742. (10.1021/acs.analchem.9b04116) 31622079

[42] Li G, Tang Z. 2014 Noble metal nanoparticle@metal oxide core/yolk-shell nanostructures as catalysts: recent progress and perspective. Nanoscale **6**, 3995-4011. (10.1039/c3nr06787d) 24622876

[43] Cui W, Xue D, Tan N, Zheng B, Jia M, Zhang W. 2018 Pt supported on octahedral Fe_3_O_4_ microcrystals as a catalyst for removal of formaldehyde under ambient conditions. Chin. J. Catal. **39**, 1534-1542. (10.1016/s1872-2067(18)63082-7)

[44] Zubir NA, Yacou C, Motuzas J, Zhang X, Diniz da Costa JC. 2014 Structural and functional investigation of graphene oxide-Fe_3_O_4_ nanocomposites for the heterogeneous Fenton-like reaction. Sci. Rep. **4**, 4594. (10.1038/srep04594) 24699690 PMC3975239

[45] Bai L, Jiang W, Sang M, Liu M, Xuan S, Wang S, Leung KC-F, Gong X. 2019 Magnetic microspheres with polydopamine encapsulated ultra-small noble metal nanocrystals as mimetic enzymes for the colorimetric detection of H_2_O_2_ and glucose. J. Biomed. Mater. Res. B Appl. Biomater. **7**, 4568-4580. (10.1039/c9tb00755e)

[46] Zheng J, Dong Y, Wang W, Ma Y, Hu J, Chen X, Chen X. 2013 *In situ* loading of gold nanoparticles on Fe_3_O_4_@SiO_2_ magnetic nanocomposites and their high catalytic activity. Nanoscale **5**, 4894-4901. (10.1039/c3nr01075a) 23624783

[47] Gao M, An P, Rao H, Niu Z, Xue X, Luo M, Liu X, Xue Z, Lu X. 2020 Molecule-gated surface chemistry of Pt nanoparticles for constructing activity-controllable nanozymes and a three-in-one sensor. Analyst **145**, 1279-1287. (10.1039/c9an01956a) 31867591

[48] Ma M, Xie J, Zhang Y, Chen Z, Gu N. 2013 Fe_3_O_4_@Pt nanoparticles with enhanced peroxidase-like catalytic activity. Mater. Lett. **105**, 36-39. (10.1016/j.matlet.2013.04.020)

[49] He Y, Wang P, Chen X, Li Y, Wei J, Cai G, Aoyagi K, Wang W. 2022 Data from: Facile preparation of Fe_3_O_4_@Pt nanoparticles as peroxidase mimics for sensitive glucose detection by a paper-based colorimetric assay. Figshare. (10.6084/m9.figshare.c.6197431) PMC9515637 36177202

